# *In vitro* and *in vivo* Assessment of Keratose as a Novel Excipient of Paclitaxel Coated Balloons

**DOI:** 10.3389/fphar.2018.00808

**Published:** 2018-07-30

**Authors:** Emily Turner, Megan Erwin, Marzieh Atigh, Uwe Christians, Justin M. Saul, Saami K. Yazdani

**Affiliations:** ^1^Department of Mechanical Engineering, University of South Alabama, Mobile, AL, United States; ^2^Department of Anesthesiology, iC42 Clinical Research and Development, University of Colorado, Aurora, CO, United States; ^3^Department of Chemical, Paper and Biomedical Engineering, Miami University, Oxford, OH, United States

**Keywords:** drug coated balloon, paclitaxel, keratose, keratin, excipient, coating, pharmacokinetics, preclinical evaluation

## Abstract

**Purpose:** Drug coated balloons (DCB) are continually improving due to advances in coating techniques and more effective excipients. Paclitaxel, the current drug choice of DCB, is a microtubule-stabilizing chemotherapeutic agent that inhibits smooth muscle cell proliferation. Excipients work to promote coating stability and facilitate paclitaxel transfer and retention at the target lesion, although current excipients lack sustained, long-term paclitaxel retention. Keratose, a naturally derived protein, has exhibited unique properties allowing for tuned release of various therapeutic agents. However, little is known regarding its ability to support delivery of anti-proliferative agents such as paclitaxel. The goal of this project was to thus demonstrate the feasibility of keratose as a DCB-coating excipient to promote the release and delivery of paclitaxel.

**Methods:** Keratose was combined with paclitaxel *in vitro* and the release kinetics of paclitaxel and keratose were evaluated through high performance liquid chromatograph-mass spectroscopy (HPLC-MS) and spectrophotometry, respectively. A custom coating method was developed to deposit keratose and paclitaxel on commercially available angioplasty balloons via an air spraying method. Coatings were then visualized under scanning electron microscopy and drug load quantified by HPLC-MS. Acute arterial transfer of paclitaxel at 1 h was assessed using a novel *ex vivo* model and further evaluated *in vivo* in a porcine ilio-femoral injury model.

**Results:** Keratose demonstrated tunable release of paclitaxel as a function of keratose concentration *in vitro*. DCB coated via air spraying yielded consistent drug loading of 4.0 ± 0.70 μg/mm^2^. Under scanning electron microscopy, the keratose-paclitaxel DCB showed uniform coverage with a consistent, textured appearance. The acute drug transfer of the keratose-paclitaxel DCB was 43.60 ± 14.8 ng/mg at 1 h *ex vivo*. These measurements were further confirmed *in vivo* as the acute 1 h arterial paclitaxel levels were 56.60 ± 66.4 ng/mg.

**Conclusion:** The keratose-paclitaxel coated DCB exhibited paclitaxel uptake and achieved acute therapeutic arterial tissue levels, confirming the feasibility of keratose as a novel excipient for DCB.

## Introduction

The use of drug eluting stents (DES) was a major breakthrough in reducing the risk of restenosis following stent-induced injury (Morice et al., 2002; Stone et al., 2004). DES utilize polymer excipients on the stent platform to deliver anti-proliferative drugs over several weeks to months (Daemen et al., 2007). Current 2nd and 3rd generation DES have significantly improved clinical success as compared to 1st generation DES, with the incidence of restenosis falling below 10% (Stone et al., 2009; Navarese et al., 2013). However, the clinical success of DES is limited to the coronary vasculature and does not translate to treatment of peripheral arterial disease (PAD) (Singh et al., 2014). Unlike the coronaries, stents in the periphery are subjected to biomechanical stress and risk of strut fracture (Scheinert et al., 2005). Fractured stents exert additional stresses on the artery wall and increase re-occlusion rates (Scheinert et al., 2005; Rits et al., 2008). Furthermore, small diameter peripheral vessels, often occluded in diabetic patients, are not conducive to stent intervention which precludes their use in below-the-knee applications. Revascularization of these arteries are essential for limb salvage in diabetic patients (Sumpio et al., 2003; Ricco et al., 2013). These limitations of DES in the treatment of PAD have revived ideas of delivering anti-proliferative drugs without the use of a metallic stent platform leading to the advent of non-stent local drug delivery devices.

Drug coated balloons (DCB) have recently emerged as a new non-stent therapeutic approach to treat PAD (Werk et al., 2008, 2012; Scheinert et al., 2014; Tepe et al., 2015a,b). DCB are angioplasty balloons directly coated with an anti-proliferative therapeutic drug and excipient (drug carrier) to aid in drug transfer. DCB were approved for the treatment of PAD in 2009 in Europe and late 2014 in the United States. During this time, there have been a wide variety of excipients used (Scheller et al., 2003a; Cortese et al., 2010; Cremers et al., 2010; Hehrlein et al., 2011; Scheinert et al., 2014; Schroeder et al., 2015). Excipients aid in many aspects of a DCB design, which includes: (1) enhancing adhesion of the drug to the balloon surface, (2) increasing stability of the drug coating during handling, (3) enhancing release of the drug coating during deployment, (4) increasing drug solvation, (5) maximizing binding of the drug coating to the vessel wall, and (6) sustaining a drug depot at the vessel surface (Cremers et al., 2010; Venkatasubbu et al., 2012; Buszman et al., 2013; Seidlitz et al., 2013; Kempin et al., 2015; Lockwood, 2015; Spectranetics, 2017). In the United States, DCB excipients include polysorbate and sorbitol, urea, polyethylene glycol (PEG), and butyryl-tri-hexyl citrate (BTHC). Other excipients used in Europe include dimethyl sulfoxide (DMSO) and shellac. Each excipient serves a different function. For example, polysorbate and PEG are known cosolvents of paclitaxel (Tarr and Yalkowsky, 1987; Adams et al., 1993) and urea acts to increase paclitaxel release at the lesion (Cremers et al., 2010). The most recently FDA-approved DCB, Spectranetics Stellarex^TM^, uses a PEG excipient shown to bind to hydroxyl apatite, a primary component of calcified atherosclerotic lesions (Venkatasubbu et al., 2012; Lockwood, 2015; Schroeder et al., 2015; Spectranetics, 2017). Though each of these excipients have led to improvements in clinical results as compared to non-coated balloon angioplasty, these outcomes hold much room for improvement (Werk et al., 2008, 2012; Cortese et al., 2010; Scheinert et al., 2014; Zeller et al., 2014; Rosenfield et al., 2015; Tepe et al., 2015a,b). There exists still a need for a more durable, longer-lasting drug carrier to elongate arterial drug delivery and further inhibit neointimal hyperplasia and restenosis. As excipients are constantly evolving and improving, the function of excipients used also needs to expand.

Keratin, an endogenous structural protein found in hair and nails, is an increasing used biomaterial that has been demonstrated in a wide variety of applications including wound healing, (Poranki and Van Dyke, 2014; Park et al., 2015) nerve repair, (Hill et al., 2011; Lin et al., 2012; Pace et al., 2014) treatment of myocardial infarction, (Shen et al., 2011) and hemostasis (Aboushwareb et al., 2009; Nunez et al., 2013; Burnett et al., 2014). Keratose, the oxidatively purified extract of keratin, has low toxicity and immunogenicity and can spontaneously forms scaffolds capable of eluting drugs/factors in a concentration-dependent manner (Hill et al., 2010; de Guzman et al., 2011; Saul et al., 2011; Burnett et al., 2013). Saul et al. (2011) demonstrated that release of ciprofloxacin from keratose is highly correlated to degradation of keratose. Ham et al. (2016) also demonstrated correlation of IGF-1 release to keratin degradation. This *in vivo* degradation is mediated solely through hydrolytic degradation as there are no known mammalian keratinases (Mogosanu et al., 2014). As such, keratins are a material of interest as drug delivery depots in tissue engineering strategies for drug delivery. However, to our knowledge, the ability to use keratose for the delivery of drugs from coatings of balloon has not previously been reported. The goal of this study was to thus evaluate the feasibility of a paclitaxel delivery via a keratose-paclitaxel drug coated balloon. Specifically, paclitaxel release was determined *in vitro* and arterial retention of paclitaxel was investigated using an *ex vivo* and a swine injury model. To our knowledge, this is the first use and evaluation of a biomaterial as an excipient to paclitaxel.

## Materials and Methods

### Chemicals and Materials

Liquid paclitaxel (6 mg/mL) was purchased from Sagent Pharmaceuticals (Schaumburg, IL, United States). Powdered paclitaxel was purchased from LC Laboratories (Woburn, MA, United States). Sterile (2 MRad gamma irradiation), lyophilized keratose was obtained from KeraNetics LLC (Winston-Salem, NC, United States) and used without further modification and under aseptic conditions. Iohexol (OMNIPAQUE^TM^ [300 mgI/mL]) was purchased from GE Healthcare (Little Chalfont, United Kingdom). Docetaxel was purchased from Tocris Bioscience (Bristol, United Kingdom). DMSO, methanol, water, acetonitrile, and formic acid were procured through Sigma-Aldrich (St. Louis, MO, United States). Absolute ethanol 200-proof was purchased from Fisher Scientific (Hampton, NH, United States).

### *In vitro* Keratose-Paclitaxel Degradation/Release

**FIGURE 1:**
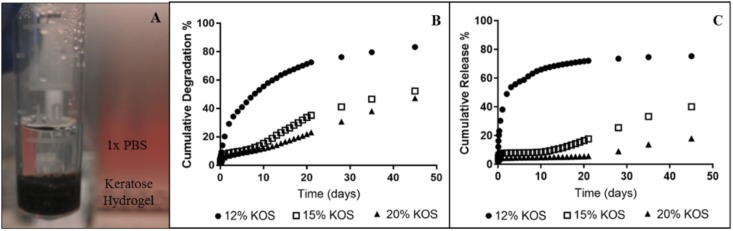
*In vitro* paclitaxel release from keratose hydrogels (one sample per each hydrogel). **(A)** Set-up for quantifying paclitaxel time-dependent release, **(B)** Cumulative degradation of keratose, and **(C)** Cumulative release of paclitaxel.

Paclitaxel-containing keratose hydrogels were formed at 12, 15, and 20% weight-to-volume ratios of keratose in phosphate buffered saline (PBS) (GE Healthcare Hyclone, Logan, UT, United States) as previously described (Turner et al., 2017). Hydrogels were injected into microcentrifuge tubes. The volume of each hydrogel (12, 15, and 20%) was 350 μL, each containing 500 μg of paclitaxel. Keratose-paclitaxel hydrogels were incubated at 37°C, 500 μL of 1× PBS was added on top of each hydrogel and at specified time points (up to 45 days) the PBS was removed for quantification and replaced with fresh PBS (**Figure 1A**).

#### Quantification of Paclitaxel From *in vitro* Release

Paclitaxel was quantified from PBS samples containing keratose by high-performance liquid chromatography tandem mass spectrometry (HPLC-MS). Prior to HPLC-MS, keratose was removed from the samples by ethanol extraction (Turner et al., 2017). A Symmetry C18, 100 Å, 3.5 μm, 100 × 2.1 mm column (Waters Corporation, Milford, MA, United States) was used for separation. Using a previously developed HPLC-MS method, paclitaxel was quantified with an LTQ Velos Orbitrap Mass Spectrometer with Electrospray Ionization (Thermo Fisher Scientific, Waltham, MA, United States) run in positive ion mode (Turner et al., 2017). Paclitaxel was detected at 854 m/z. The internal standard, docetaxel, was detected at 808 m/z (Turner et al., 2017).

#### Quantification of Keratose

Keratose samples were diluted at 1:1, 1:2, 1:4, and 1:6 in triplicate. Following the manufacturer’s protocol, keratose was quantified in triplicate using the DC Protein Assay (Bio-Rad Laboratories, Hercules, CA, United States). Absorbance was measured with a SpectraFluor microplate reader (Tecan, Männedorf, Switzerland) at 750 nm.

### Balloon Coating

**FIGURE 2:**
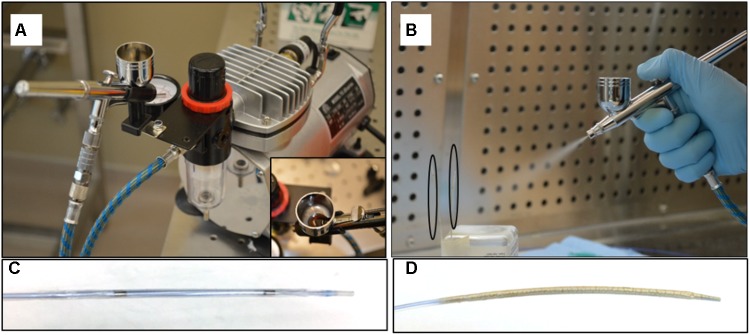
Air spray coating. **(A)** Master Airbrush coating system with TC-20 compressor, and 0.3 mm tip G22 high performance airbrush, **(B)** Coating of paclitaxel and keratose onto balloon surface – balloons outlined by black ovals. **(C)** Non-coated angioplasty balloon. **(D)** Keratose-paclitaxel coated drug coated balloon.

Paclitaxel was prepared by dissolving in absolute ethanol followed by sonication at a final concentration of 40 mg/mL. Keratose solution was prepared by dissolving lyophilized keratose in iohexol at a 6% weight-to-volume ratio in iohexol. An in-house air spray coating method, via a Master Airbrush (TCP Global, San Diego, CA, United States), was used to deposit keratose and paclitaxel in a layered approach (**Figure 2**). Briefly, up to four balloons were mounted upright on a coating stand. Underneath a backdraft hood, keratose, paclitaxel, and keratose were air-spray coated in succession by loading a predetermined volume in the airbrush fluid cup. Moving the airbrush up and down the balloon(s) were coated on four sides to achieve a uniform coating circumferentially. For balloons sized 6 × 30 mm and 4.5 × 40 mm (Abbott Vascular, Abbott Park, IL, United States) 1 mL of keratose was used for each keratose layer and 7 mL of paclitaxel [40 mg/mL] for the paclitaxel layer. The layers were sprayed consecutively with no drying time between layers. The sprayer was cleaned in between each solution and following the final layer, the coated balloons were dried for a minimum of 1 h. Balloons were then sterilized by UV irradiation overnight.

### Scanning Electron Microscopy

Keratose-paclitaxel coated balloon segments and commercially available DCBs (Lutonix-Bard Peripheral Vascular, Tempe, AZ, United States and SeQuent Please, B. Braun Melsungen AG, Berlin, Germany) were cut from the catheter, mounted on studs, and sputter coated with gold palladium. A Quanta 250 scanning electron microscope with xT Microscope Control software (FEI, Hillsboro, OR, United States) was used for imaging.

### Arterial Deployment

**FIGURE 3:**
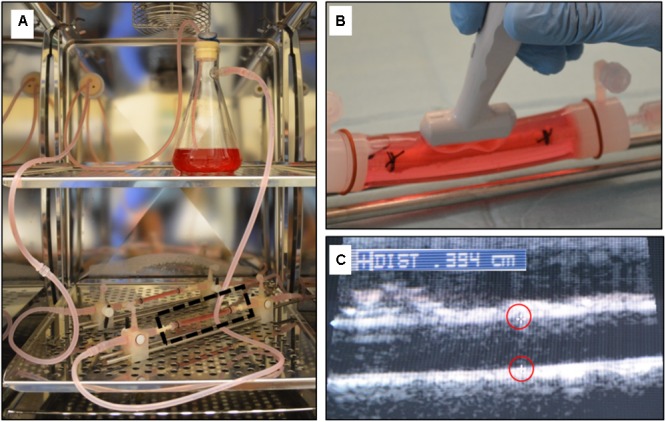
*Ex vivo* bioreactor system. **(A)** A gear pump generated pulsatile through explanted arteries within the CO2 incubator. **(B)** The explanted artery is bathed in cell culture media and an ultrasound probe can be directly placed on the outer sleeve of the bioreactor housing to measure the diameter. **(C)** Ultrasound image showing the inner diameter of the explanted artery (markers outlined in red).

Porcine carotid arteries from large pigs (250–350 lbs.) were harvested from a local abattoir. The arteries were transferred in sterile PBS with 1% antibiotic-antimitotic (Gibco, Grand Island, NY, United States), rinsed in sterile PBS in a culture hood and trimmed. Eight cm long segments were cut and tied with suture onto fittings within a bioreactor setup (**Figure 3**). The vessels were subjected to pulsatile flow as defined by a custom LabVIEW program as previously described (Atigh et al., 2017). The flow medium was made up of Dulbecco’s modified eagle’s medium containing 10% fetal bovine serum and 1% antibiotic-antimycotic. Prior to deployment, vessel diameter was evaluated by ultrasound and vessels were denuded with balloon angioplasty (**Figure 3**). Keratose-paclitaxel coated DCB were deployed for 2 min at a 10–20% overstretch. At 1 h, flow was ceased and the treated portion of the vessel was removed. Excised vessels were flash frozen, stored at –80°C and shipped on dry ice to iC42 Clinical Research and Development (Aurora, CO, United States) for quantification of arterial paclitaxel.

### Residual Paclitaxel Following Deployment

Paclitaxel remaining on the balloon following *ex vivo* and *in vivo* deployment was quantified by sonication of the balloon in ethanol for 30 min and subsequent HPLC-MS.

### Quantification of Paclitaxel Tissue Concentrations

Paclitaxel tissue concentrations were quantified using a validated, previously described high-performance liquid chromatography (HPLC)-electrospray ionization- tandem mass spectrometry system (LC-MS/MS) (Zhang et al., 2005). The system was made up of a series 1260 HPLC system (Agilent Technologies, Santa Clara, CA, United States) and an AB Sciex 5000 triple-stage quadrupole mass spectrometer (AB Sciex, Concord, ON, Canada). Paclitaxel-D_5_ was purchased from Toronto Research Chemicals (Toronto, ON, Canada) to be used as the internal standard. Untreated pig arteries were used to prepare calibration curves consisting of paclitaxel concentrations ranging from 0.5 to 100 ng/mL (Atigh et al., 2017).

Briefly, 100 μL of sample was injected onto a 4.6 ⋅ 12.5 mm 5 μm extraction column (Eclipse XDB C-8, 5 μm particle size, Agilent Technologies, Palo Alto, CA, United States) with a flow rate of 3 mL/min and a temperature of 65°C. Samples were then washed with a mobile phase of 15% methanol and 85% 0.1% formic acid. The flow was 3 mL/min and the temperature for the extraction column was set to 65°C. After 1 min of washing the samples with mobile phase (15% methanol; 85% 0.1% formic acid), the analytes were then back-flushed from the extraction column onto a 150 ⋅ 4.6 mm C8, analytical column (Zorbax XDB C8, 3.5 μm particle size, Agilent Technologies, Palo Alto, CA, United States). Paclitaxel was detected in the positive multi-reaction mode using the following ion transitions: m/z = 876.6 [M+Na]^+^ → 308.2. The internal standard, Paclitaxel-D_5_, was detected using the transition m/z = -881.6 [M+Na]^+^ → 313.1.

### Porcine Ilio-Femoral Injury Model

This study was approved by the Institutional Animal Care and Use Committee (IACUC) and conformed to the current Guide for the Care and Use of Laboratory Animals. Four female pigs (12.3–14.1 kg) were anesthetized and the right carotid artery was exposed under a sterile field. The caudal end of the right carotid artery was tied-off. Using micro scissors, a small incision was made to the right carotid artery and a 6 French (F) guide sheath was inserted. A NITREX^TM^ 0.014 guidewire (ev3 Inc., Plymouth, MN, United States) was inserted and, under fluoroscopic guidance, endothelial denudation using a 4 × 12 mm angioplasty balloon catheter (Abbot Vascular, Abbott Park, IL, United States) was performed to the left and right iliac arteries. Following denudation, 4.5 mm × 40 mm keratose-paclitaxel DCB were tracked to each of the iliac arteries and deployed for 2 min. Antiplatelet therapy consisted of aspirin (40 mg/day) given orally 24 h before catheterization with continued dosing throughout the in-life phase of the study, while single-dose intra-arterial heparin (150 IU/kg) and lidocaine were administered at the time of catheterization. Animals were anesthetized and euthanized by intravenous Fatal-Plus (Vortech Ltd., Dearborn, MI, United States) injection (85–150 mg/kg) at 1 h. Treated segments were excised following geographic landmarks determined by angiography. Excised arteries were stored at -80°C and, as aforementioned, shipped on dry ice to the bioanalytical laboratory for analysis (iC42 Clinical Research and Development, Aurora, CO, United States).

### Statistical Analysis

The data were presented as mean ± standard deviation. Data were compared with the Student *t*-test using GraphPad Prism 7 (GraphPad Software, La Jolla, CA, United States). A value of *p* ≤ 0.05 was considered statistically significant.

## Results

### Paclitaxel Release From Keratose Hydrogels

Centrifuge tubes were seeded with a mixture of keratose at concentrations of 12 to 20% (2 per each concentration; **Figure 1A**). Total paclitaxel was constant for all tubes, 500 μg each. Paclitaxel and keratose measurements were performed from 1 h to 45 days. To measure paclitaxel from keratose hydrogels, samples were diluted in PBS and read with a SpectraFluor plate reader. An HPLC-MS method developed by our group was used to quantify paclitaxel (Turner et al., 2017). Briefly, keratose was extracted from all samples by ethanol extraction with an extraction efficiency of 93.0 ± 3.4% (Turner et al., 2017). The method was validated by an intra- and inter-day precision of 4.3 and 7.9%, respectively. This precision is consistent with values for quantification of paclitaxel from plasma samples (1.04 and 12.3% RSD; Gardner et al., 2008; Lian et al., 2013; Tekade et al., 2013; Rezazadeh et al., 2015) and is effective in measuring paclitaxel concentrations from aqueous, keratin-containing samples.

Keratose degraded more quickly at a lower concentration of keratose and more slowly at a higher concentration of keratose (**Figure 1B**). The 12% keratose-paclitaxel degraded most rapidly with over 80% degradation at 45 days. The slower degradation was shown by the 20% keratose-paclitaxel hydrogel which degraded only 47% at 45 days. Paclitaxel release from keratose hydrogels showed similar gradients wherein the higher concentration of keratose released paclitaxel more slowly and the lower concentration of keratose released paclitaxel more rapidly (**Figure 1C**). The results confirm the ability of keratose to modulate release of paclitaxel *in vitro*.

### Scanning Electron Microscopy

**FIGURE 4:**
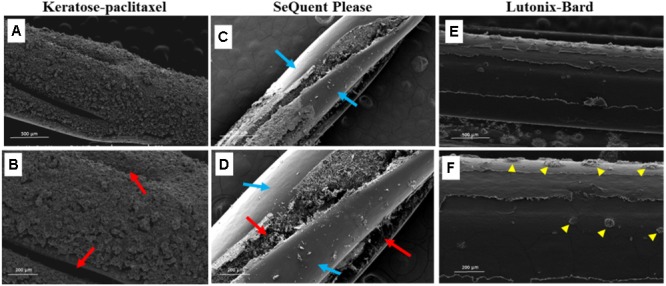
Scanning electron micrograph of various drug coated balloons (DCB). **(A,B)** SEM images of keratose-paclitaxel DCB showing uniform textured coating. Red arrows indicate folding in the balloon. **(C,D)** SEM images of Sequent Please DCB showing a mixture of bare (non-coated) and textured coating. Regions of sparse coating exposing the surface of the balloon angioplasty is shown in blue arrows. Textured coating mostly notable within the folds of the balloon (red arrows). **(E,F)** SEM images of Lutonix-Bard DCB showing uniform smooth coating. Yellow arrow heads indicated circular droplet textures.

**Figure 4** displays SEM surface images of the keratose-paclitaxel, Sequent Please and Lutonix-Bard DCB. The keratose-paclitaxel and Lutonix-Bard DCB surfaces shows uniform coating, however, the Sequent Please displayed areas of sparse coating. Overall, the surface of the keratose-paclitaxel and Sequent Please show a rougher texture as compared to the Lutonix-Bard. The textured surface in the Sequent Please DCB can be observed on the surface and in the balloon folds region, whereas in the keratose-paclitaxel DCB the textured surface is uniformly distributed on the surface. The Lutonix-Bard DCB surface overall is very smooth (non-textured) with small circular rough patterns.

### *Ex vivo* and *in vivo* Assessment of Keratose DCB

Commercially available non-coated angioplasty balloons were successfully coated with a keratose-paclitaxel mixture (**Figure 2**). For the *ex vivo* model, DCB were inserted through a 5 Fr sheath into a closed-circulatory system with physiological parameters (put pressure and flow rates). Following diameter ultrasound imaging of the harvested arteries, DCB were deployed at a balloon-to-artery ratio ranging from 1.1 to 1.2:1.0 for 1 min. Treated sections were clearly marked during inflation on the bioreactor, and following 1 h, treated section of the arteries were removed and analyzed for arterial tissue paclitaxel concentration (see **Table 1**). Pharmacokinetic evaluation of the *ex vivo* arteries demonstrated a paclitaxel concentration at 1 h was 43.60 ± 14.8 ng/mg (*n* = 4). Residual paclitaxel, remaining paclitaxel on the DCB following deployment *ex vivo*, was 10.51 ± 9.8% (*n* = 4).

**Table 1:** Comparison of *ex vivo* and *in vivo* arterial paclitaxel levels following keratose-paclitaxel DCB delivery.

Testing Model	Paclitaxel (ng/mg)	*p*-value
*Ex vivo*	43.60±14.8	0.71
*In vivo*	56.60±66.4	


**FIGURE 5:**
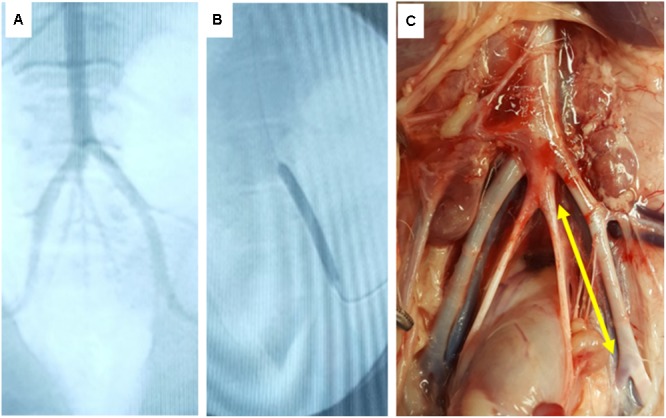
*In vivo* deployment of DCB. **(A)** Angiographic image of the ilio-femoral artery of the *in vivo* pig model. **(B)** Angiographic image of the DCB in its deployed state. **(C)** Gross image of the explant site of the treated arterial segment (yellow arrow indicates treatment site).

To further demonstrate the keratose coating to delivery paclitaxel to arteries, and to confirm the *ex vivo* results, keratose-paclitaxel DCB were deployed in a swine femoral injury model (**Figure 5**). The target balloon-to-artery ratio was 1.1–1.2:1.0 with an inflation time of 1 min. The time from insertion into the blood stream to the deployment site was less than 1 min. All treated arteries were free of dissection or any acute thrombotic event as determined by angiography. Following 1 h, treated arteries were explanted and analyzed by pharmacokinetics. The exact location of treatment was identified by anatomical landmarks (**Figure 5**). Paclitaxel drug level at 1 h was 56.6 ± 66.4 ng/mg (*n* = 3). Residual paclitaxel of DCB following deployment *in vivo* was 12.77 ± 9.4% (*n* = 3). In comparing *in vivo* and *ex vivo* arterial paclitaxel results, similar arterial paclitaxel levels were measured at 1 h (56.6 ± 66.4 ng/mg vs. 43.60 ± 14.8 ng/mg, *p* = 0.71).

## Discussion

This study evaluates, for the first time, the use of keratose as an excipient of paclitaxel for development of a novel DCB. The ability of keratose to deliver/release paclitaxel in a keratose-concentration dependent manner was first confirmed in an *in vitro* setup and quantified by a previously validated, in-house HPLC-MS method (Turner et al., 2017). A custom coating method resulted in uniform coating of angioplasty balloons with a keratose-paclitaxel coating. An *ex vivo* model demonstrated the ability of the keratose-paclitaxel DCB to transfer drug to arterial tissue at 1 h. Pharmacokinetic analysis of treated pig arteries *in vivo* confirmed *ex vivo* results and confirmed the presence of paclitaxel at therapeutic levels. Overall, these results demonstrate the feasibility to deliver paclitaxel via a keratose-paclitaxel DCB.

Non-stent delivery platforms, such as DCB, are seen as a next generation approach to overcome the limitations of stents in the treatment of PAD (Cortese et al., 2016). Stents in the periphery are subject to biomechanical stress increasing the risk of fracture, leading to device failure (Scheinert et al., 2005; Nikanorov et al., 2008; Davaine et al., 2013). The primary advantage of DCB is the uniformity and short-term transfer of drug to the luminal surface without the need of a stent platform and a polymer carrier (Scheller et al., 2004). The success of DCB in PAD treatment has been shown in several randomized clinical trials, demonstrating superiority of this technology over standard non-coated balloons (i.e., plain old balloon angioplasty, POBA) in the treatment of femoropopliteal (above-the-knee) arteries (Werk et al., 2012; Scheinert et al., 2014; Rosenfield et al., 2015; Tepe et al., 2015a). To date, most clinical studies demonstrate the benefit of DCB up to 12 months in relatively short lesions, with few studies reporting follow-ups longer than 1 year (Tepe et al., 2008, 2015a; Werk et al., 2012; Micari et al., 2013; Scheinert et al., 2014; Rosenfield et al., 2015; Schroeder et al., 2015). In one of the only long-term (2 year) real-world registries, results suggest that DCB are safe and effective in delaying rather than preventing restenosis in long, complex lesions of the femoropopliteal (above-the-knee) region (Schmidt et al., 2016). For the treatment of infrapopliteal (below-the-knee) disease, DCB have shown to be less effective with no clinical benefit over standard non-coated balloons (Zeller et al., 2014; Zhang et al., 2017). These clinical studies confirm the benefits of DCB clinically, however, improvements to DCB are needed to recover long-term clinical results. One such target for long-term clinical success is long-term paclitaxel retention at the artery by careful selection of excipients to modulate such retention.

Current generation of DCB primarily use the anti-proliferative drug paclitaxel. Paclitaxel, which is extracted from pacific yew bark, inhibits cell division by binding to growing microtubules (Rowinsky, 1993). Used as a cancer therapeutic since 1992, paclitaxel is active against ovarian cancer, breast cancer, Kaposi’s sarcoma, and lung cancer (Rowinsky, 1993; Oberhoff et al., 2002). More recently, paclitaxel has been used as an anti-restenotic in cardiovascular interventions such as DES and DCBs to inhibit smooth muscle cell proliferation – a primary contributor to restenosis (Suffness, 1995; Axel et al., 1997; Haehnel et al., 1998; Loh and Waksman, 2012). The most advantageous aspect of paclitaxel as compared to other currently available anti-proliferative agents is that it is highly potent and has shown to be effective in the short, single dose approach (Speck et al., 2012). However, paclitaxel is highly lipophilic and nearly insoluble in water, and has shown to be more effective when delivered with excipients as compared to paclitaxel alone in treatment of vascular disease (Scheller et al., 2003a,b; Atigh et al., 2017).

The role of excipients in DCB serves many functions. First, excipients aid in adherence of the coating to the balloon itself. Upon arrival at the lesion, excipients aide in release of the coating from the balloon, deposition of the coating at the vessel surface, and uptake of the drug into the surrounding tissue. Finally, the excipient/drug must stay in place at the lesion, upholding the drug deposit and continuing drug elution into the tissue. DCBs are susceptible to loss of the drug coating during balloon tracking and deployment (Kelsch et al., 2011). Additionally, drug deposited at the vessel post-deployment is subjected to wash-off into the blood stream, further decreasing the long-term drug residency. Therefore, improved excipients to paclitaxel are crucial in decreasing drug loss during tracking and wash-off into the blood stream thereby improving drug residency and success of treatment. Here, keratose demonstrated a tunable release of paclitaxel as a function of keratose concentration *in vitro*. The novelty of tunable release of paclitaxel as a function of keratose lies in the ability to adjust keratose concentration to facilitate paclitaxel release in a time-dependent fashion relative to the restenotic cascade. It is hypothesized that this release is based on encapsulation or electrostatic forces of paclitaxel within the keratose scaffold (Saul et al., 2011). This tuned-release ability of keratose has also been demonstrated previously in antibiotic delivery and has potential in further applications where release kinetics need to be adjusted (Saul et al., 2011). Scaffolds made by lyophilized keratose hydrogels have been shown to contain cylindrical casts made up of isolated and interconnected pores (de Guzman et al., 2011). Further studies are warranted to determine the precise mechanism of paclitaxel release.

Keratin, the non-oxidized form of keratose, have shown to be safe in a variety of biomedical applications including skin (Poranki et al., 2014), hemostasis (Burnett et al., 2013), bone (Dias et al., 2010), nerve regeneration (Sierpinski et al., 2008) and in the use of therapeutic delivery including antibiotics (Saul et al., 2011) and recombinant human bone morphogenetic protein (de Guzman et al., 2013). In a recent paper by Han et al., the degradation of keratin did not increase levels of toxicity due to changes in the protein or due to the presence of residual iodoacetamide (Han et al., 2015). For the *in vitro* studies, we examined paclitaxel release from keratose concentrations varying from 12 to 20%. This was done primarily to determine correlation between paclitaxel release and the rate of keratose degradation based on previously reported methods (Saul et al., 2011; Ham et al., 2016). And although the *in vitro* studies did confirm the ability of keratose to modulate release of paclitaxel, for balloon coating purposes, the keratose concentration was reduced to 6%. This deviation of concentration was necessary as the 6% keratose concentration did not gel, and thus remained in liquid form enabling us to successfully spray coat our uncoated balloons and examine the feasibility of paclitaxel transfer of a keratose-paclitaxel coated balloon. Specifically, we wanted to determine if the arterial paclitaxel levels following delivery was within the therapeutic target ranging from 0.01 to 100 ng/mg (paclitaxel amount/weight of arterial tissue; Axel et al., 1997; Buszman et al., 2013). Our *in vivo* results demonstrated arterial paclitaxel levels of 56.6 ± 66.4 ng/mg (*n* = 3) at 1 h. These results were similar to preclinical evaluation of a FDA-approved DCB with paclitaxel levels of 58.8 ± 54.2 ng/mg at 1 h (Yazdani et al., 2014). Angiography showed no sign of thrombus following DCB deployment. It is important to note keratose, extracted from keratin via oxidative extraction, used in these studies is known to be non-thrombogenic (de Guzman et al., 2011). However, reductively extracted keratin (known as kerateine) has an entirely different effect in that it is thrombogenic (Hill et al., 2010). Thus, it is not the primary protein sequence of keratin that leads to the observed effects, but rather a combination of properties of the amino acids in the primary protein structure based on its method of extraction and the properties of the keratin solutions or hydrogels. However, the properties of the hydrogels are indeed due largely to the primary amino acid properties including isoelectric point and ability to form covalent crosslinks via thiol groups. A salient feature of keratins is the presence of a large number of cysteine residues within the primary sequence. Thiol groups on cysteine amino acid residues become available for disulfide crosslinking in kerateine obtained by oxidative extraction but not in keratose where oxidative extraction leads to sulfonic acid groups on cysteine residues.

Pharmacokinetic evaluation is accepted as the gold standard method to evaluate DCB (Melder, 2012; Granada et al., 2014; Yazdani et al., 2014; Fernandez-Parra et al., 2015; Gongora et al., 2015; Spectranetics, 2017). The current paradigm for evaluating DCB is relatively non-existent prior to animal testing. Bench testing of non-stent devices (such as DCB and perfusion catheters) are confined to mechanical testing of the balloon (burst pressure, fatigue), tracking and particulate matter, and biocompatibility of the drug and carrier using cell culture and static techniques (Bandomir et al., 2015; Kaule et al., 2015; Anderson et al., 2016). *In vitro*, the total drug coated on a balloon can be quantified as well as elution from the balloon into a saline bath of controlled volume, however, the transfer of the drug from the device to an artery (arterial uptake and retention) is lacking. It is these limitations that drug delivery parameters are tested and verified *in vivo* by removing treated vessels at several time points to determine the acute transfer of drug and drug retention. Although these trials are essential in determining drug delivery, they are often very long, costly, and utilize a large number of animals. The developed *ex vivo* bioreactor system described here permits the use of explanted living native pig arteries to be used as the test section. Previously we have shown that harvested swine arteries maintain functionality up to 7 days *ex vivo* (Yazdani and Berry, 2009). This is particularly important because there are currently no man-made vessels that can duplicate the cellular organization, structure, and elasticity of the native artery. Furthermore, the explanted arteries can mimic the acute transfer of the drug from the device, which is the most significant aspect of DCB platforms.

Other benefits of our *ex vivo* system include the ability to evaluate any commercially available vascular device, running multiple experiments simultaneously, obtaining the results in days, and the low cost associated with performing *ex vivo* experiments as compared to *in vivo* experiments. Additionally, due to the design of the system, the inner diameter of the explanted arteries can be monitored and measured using ultrasound (**Figure 3**). This is particularly important for deployment of balloons and stents as they are designed to be inflated in respect to the vessel diameter. Under deployment or over deployment of these devices directly affect and can alter drug transfer to the artery.

The keratose-paclitaxel DCBs lacked coating within the balloon folds. To achieve coating within the folds of the balloon, the balloon must be coated in the deployed state and then carefully refolded. Refolding of the coated balloon requires use of a very costly piece of equipment which precisely refolds the balloons. Due to the lack of coating within the folds of the balloons in this study the drug distribution following deployment is not uniform circumferentially. Despite this limitation, arterial transfer and uptake of paclitaxel can still be quantified. Pharmacokinetics *in vivo* were evaluated in healthy and not diseased arteries. It was decided not to use a disease model because this is the first instance that keratose has been used as an excipient for vascular drug delivery and was therefore a proof-of-concept study. It was crucial to test the keratose-paclitaxel balloon in a non-disease model at this time because the complexities of a disease model may mask keratose-specific effects. In addition, no off-the shelf products were evaluated for a side by side comparison. Longer time points *in vivo* should be assessed to evaluate long-term pharmacokinetics and histology of the keratose-paclitaxel balloon. Some limitations of our system include the fact that culture medium was the working fluid rather than whole blood. The generated *ex vivo* flow conditions did not duplicate *in vivo* conditions. Another limitation of the current studies includes the inflicted damage to cells during the carotid artery harvest. In spite of these limitations, evaluation of the pharmacokinetics can be accomplished. Off-the-shelf vascular devices can be inserted, conditioned at physiological mechanical conditions in a native artery *ex vivo*, and evaluated for drug pharmacokinetics.

To conclude, this study provides the first evidence of the use of keratose as a novel excipient to coat DCB. Pharmacokinetic studies demonstrated therapeutic levels of paclitaxel at 1 h in arteries treated with a keratose-paclitaxel coating *ex vivo* and *in vivo*. Novel excipients, such as keratose, can potentially improve upon the focus of current excipients which are designed to increase solubility of paclitaxel and penetration of the drug to long-term sustained drug. Further studies are warranted to demonstrate such benefits of keratose. Safety of the keratose-paclitaxel DCB can be assessed by delivery of 2× or 4× dose *in vivo* followed by histological evaluation. Overall, this approach has the potential to improve interventional outcomes and quality of life of millions of patients suffering with PAD.

## Author Contributions

SY, ET, and JS contributed to the conception and design of the study. ET, MA, and ME organized the database. SY, ET, MA, ME, UC, and JS performed the data analysis. SY and ET performed the statistical analysis. SY and ET wrote the first draft of the manuscript. All authors contributed to the manuscript revision, read, and approved the submitted version.

## Conflict of Interest Statement

SY serves on the Scientific Advisory Board of Advanced Catheter Therapies and Toray Industries and has received grant support from Advanced Catheter Therapies, Toray Industries and Lutonix, Inc., and is a shareholder in Advanced Catheter Therapies. JS has received previous funding from KeraNetics, LLC. The remaining authors declare that the research was conducted in the absence of any commercial or financial relationships that could be construed as a potential conflict of interest.

## References

[B1] Aboushwareb T. Eberli D. Ward C. Broda C. Holcomb J. Atala A. (2009). A keratin biomaterial gel hemostat derived from human hair: evaluation in a rabbit model of lethal liver injury. *J. Biomed. Mater. Res. B Appl. Biomater.* 90 45–54. 10.1002/jbm.b.31251 18988274

[B2] Adams J. D. Flora K. P. Goldspiel B. R. Wilson J. W. Finley R. (1993). Taxol^®^: a history of pharmaceutical development and current pharmaceutical concerns. *J. Natl. Cancer Inst. Monogr.* 15 141–147. 7912520

[B3] Anderson J. A. Lamichhane S. Remund T. Kelly P. Mani G. (2016). Preparation, characterization, in vitro drug release, and cellular interactions of tailored paclitaxel releasing polyethylene oxide films for drug-coated balloons. *Acta Biomater.* 29 333–351. 10.1016/j.actbio.2015.09.036 26432441

[B4] Atigh M. K. Turner E. A. Christians U. Yazdani S. K. (2017). The use of an occlusion perfusion catheter to deliver paclitaxel to the arterial wall. *Cardiovasc. Ther.* 35:e12269. 10.1111/1755-5922.12269 28445625 PMC5511576

[B5] Axel D. I. Kunert W. Goggelmann C. Oberhoff M. Herdeg C. Kuttner A. (1997). Paclitaxel inhibits arterial smooth muscle cell proliferation and migration in vitro and in vivo using local drug delivery. *Circulation* 96 636–645. 10.1161/01.CIR.96.2.636 9244237

[B6] Bandomir J. Kaule S. Schmitz K.-P. Sternberg K. Petersen S. Kragl U. (2015). Usage of different vessel models in a flow-through cell: in vitro study of a novel coated balloon catheter. *RSC Adv.* 5 11604–11610. 10.1039/C4RA12524J

[B7] Burnett L. R. Rahmany M. B. Richter J. R. Aboushwareb T. A. Eberli D. Ward C. L. (2013). Hemostatic properties and the role of cell receptor recognition in human hair keratin protein hydrogels. *Biomaterials* 34 2632–2640. 10.1016/j.biomaterials.2012.12.022 23340195

[B8] Burnett L. R. Richter J. G. Rahmany M. B. Soler R. Steen J. A. Orlando G. (2014). Novel keratin (KeraStat) and polyurethane (Nanosan(R)-Sorb) biomaterials are hemostatic in a porcine lethal extremity hemorrhage model. *J. Biomater. Appl.* 28 869–879. 10.1177/0885328213484975 23594681

[B9] Buszman P. P. Tellez A. Afari M. Cheng Y. Conditt G. B. Mcgregor J. C. (2013). Stent healing response following delivery of paclitaxel via durable polymeric matrix versus iopromide-based balloon coating in the familial hypercholesterolaemic swine model of coronary injury. *EuroIntervention* 9 510–516. 10.4244/EIJV9I4A82 23965356

[B10] Cortese B. Granada J. F. Scheller B. Schneider P. A. Tepe G. Scheinert D. (2016). Drug-coated balloon treatment for lower extremity vascular disease intervention: an international positioning document. *Eur. Heart J.* 37 1096–1103. 10.1093/eurheartj/ehv204 26009594

[B11] Cortese B. Micheli A. Picchi A. Coppolaro A. Bandinelli L. Severi S. (2010). Paclitaxel-coated balloon versus drug-eluting stent during PCI of small coronary vessels, a prospective randomized clinical trial. *Heart* 96 1291–1296. 10.1136/hrt.2010.195057 20659948

[B12] Cremers B. Clever Y. Schaffner S. Speck U. Böhm M. Scheller B. (2010). Treatment of coronary in-stent restenosis with a novel paclitaxel urea coated balloon. *Minerva Cardioangiol.* 58 583–588. 20948504

[B13] Daemen J. Wenaweser P. Tsuchida K. Abrecht L. Vaina S. Morger C. (2007). Early and late coronary stent thrombosis of sirolimus-eluting and paclitaxel-eluting stents in routine clinical practice: data from a large two-institutional cohort study. *Lancet* 369 667–678. 10.1016/S0140-6736(07)60314-6 17321312

[B14] Davaine J. M. Querat J. Guyomarch B. Brennan M. A. Costargent A. Chaillou P. (2013). Incidence and the clinical impact of stent fractures after primary stenting for TASC C and D femoropopliteal lesions at 1 year. *Eur. J. Vasc. Endovasc. Surg.* 46 201–212. 10.1016/j.ejvs.2013.05.010 23773773

[B15] de Guzman R. C. Merrill M. R. Richter J. R. Hamzi R. I. Greengauz-Roberts O. K. Van Dyke M. E. (2011). Mechanical and biological properties of keratose biomaterials. *Biomaterials* 32 8205–8217. 10.1016/j.biomaterials.2011.07.054 21835462

[B16] de Guzman R. C. Saul J. M. Ellenburg M. D. Merrill M. R. Coan H. B. Smith T. L. (2013). Bone regeneration with BMP-2 delivered from keratose scaffolds. *Biomaterials* 34 1644–1656. 10.1016/j.biomaterials.2012.11.002 23211447

[B17] Dias G. J. Peplow P. V. Mclaughlin A. Teixeira F. Kelly R. J. (2010). Biocompatibility and osseointegration of reconstituted keratin in an ovine model. *J. Biomed. Mater. Res. A* 92 513–520. 10.1002/jbm.a.32394 19213058

[B18] Fernandez-Parra R. Laborda A. Lahuerta C. Lostale F. Aramayona J. De Blas I. (2015). Pharmacokinetic study of paclitaxel concentration after drug-eluting balloon angioplasty in the iliac artery of healthy and atherosclerotic rabbit models. *J. Vasc. Interv. Radiol.* 26 1380–1387.e1. 10.1016/j.jvir.2015.05.022 26190185

[B19] Gardner E. R. Dahut W. Figg W. D. (2008). Quantitative determination of total and unbound paclitaxel in human plasma following Abraxane treatment. *J. Chromatogr. B Analyt. Technol. Biomed. Life Sci.* 862 213–218. 10.1016/j.jchromb.2007.12.013 18191625 PMC2259285

[B20] Gongora C. A. Shibuya M. Wessler J. D. Mcgregor J. Tellez A. Cheng Y. (2015). Impact of paclitaxel dose on tissue pharmacokinetics and vascular healing: a comparative drug-coated balloon study in the familial hypercholesterolemic swine model of superficial femoral in-stent restenosis. *JACC Cardiovasc. Interv.* 8 1115–1123. 10.1016/j.jcin.2015.03.020 26117470

[B21] Granada J. F. Stenoien M. Buszman P. P. Tellez A. Langanki D. Kaluza G. L. (2014). Mechanisms of tissue uptake and retention of paclitaxel-coated balloons: impact on neointimal proliferation and healing. *Open Heart* 1:e000117. 10.1136/openhrt-2014-000117 25332821 PMC4189287

[B22] Haehnel I. Alt E. Resch A. Märkl A. Stemberger A. Schömig I. (1998). Local growth inhibitory effect of paclitaxel released by a biodegradable stent coating on vascular smooth muscle cells [abstract no. 1114-102]. *J. Am. Coll. Cardiol.* 31:278A 10.1016/S0735-1097(97)84916-9

[B23] Ham T. R. Lee R. T. Han S. Haque S. Vodovotz Y. Gu J. (2016). Tunable keratin hydrogels for controlled erosion and growth factor delivery. *Biomacromolecules* 17 225–236. 10.1021/acs.biomac.5b01328 26636618 PMC5565161

[B24] Han S. Ham T. R. Haque S. Sparks J. L. Saul J. M. (2015). Alkylation of human hair keratin for tunable hydrogel erosion and drug delivery in tissue engineering applications. *Acta Biomater.* 23 201–213. 10.1016/j.actbio.2015.1005.1013 25997587 PMC4522204

[B25] Hehrlein C. Richardt G. Wiemer M. Schneider H. Naber C. Hoffmann E. (2011). Description of Pantera Lux paclitaxel-releasing balloon and preliminary quantitative coronary angiography (QCA) results at six months in patients with coronary in-stent restenosis. *EuroIntervention* 7(Suppl. K) K119–K1124. 10.4244/EIJV7SKA20 22027721

[B26] Hill P. Brantley H. Van Dyke M. (2010). Some properties of keratin biomaterials: kerateines. *Biomaterials* 31 585–593. 10.1016/j.biomaterials.2009.09.076 19822360

[B27] Hill P. S. Apel P. J. Barnwell J. Smith T. Koman L. A. Atala A. (2011). Repair of peripheral nerve defects in rabbits using keratin hydrogel scaffolds. *Tissue Eng. Part A* 17 1499–1505. 10.1089/ten.TEA.2010.0184 21275820

[B28] Kaule S. Minrath I. Stein F. Kragl U. Schmidt W. Schmitz K. P. (2015). Correlating coating characteristics with the performance of drug-coated balloons–a comparative in vitro investigation of own established hydrogel- and ionic liquid-based coating matrices. *PLoS One* 10:e0116080. 10.1371/journal.pone.0116080 25734818 PMC4348426

[B29] Kelsch B. Scheller B. Biedermann M. Clever Y. P. Schaffner S. Mahnkopf D. (2011). Dose response to Paclitaxel-coated balloon catheters in the porcine coronary overstretch and stent implantation model. *Invest. Radiol.* 46 255–263. 10.1097/RLI.0b013e31820577df 21285890

[B30] Kempin W. Kaule S. Reske T. Grabow N. Petersen S. Nagel S. (2015). In vitro evaluation of paclitaxel coatings for delivery via drug-coated balloons. *Eur. J. Pharm. Biopharm.* 96 322–328. 10.1016/j.ejpb.2015.08.010 26318979

[B31] Lian H. Sun J. Zhang T. (2013). A rapid and sensitive determination of paclitaxel in rat plasma by UPLC-MS/MS method: application to a pharmacokinetic study. *Asian J. Pharm. Sci.* 8 199–205. 10.1016/j.jchromb.2015.11.056 26684720

[B32] Lin Y. C. Ramadan M. Van Dyke M. Kokai L. E. Philips B. J. Rubin J. P. (2012). Keratin gel filler for peripheral nerve repair in a rodent sciatic nerve injury model. *Plast. Reconstr. Surg.* 129 67–78. 10.1097/PRS.0b013e3182268ae0 22186500

[B33] Lockwood N. (2015). “Drug delivery to the vessel wall: coated balloons and the role of the excipient”, in *2015 BioInterface Workshop and Symposium* Scottsdale, AZ.

[B34] Loh J. P. Waksman R. (2012). Paclitaxel drug-coated balloons: a review of current status and emerging applications in native coronary artery de novo lesions. *JACC Cardiovasc. Interv.* 5 1001–1012. 10.1016/j.jcin.2012.08.005 23078727

[B35] Melder R. J. (2012). *IN/PACT Drug-Eluting Balloon: Technology and Pre-clinical Findings.* Paris: EUROPCR.

[B36] Micari A. Cioppa A. Vadala G. Castriota F. Liso A. Marchese A. (2013). 2-year results of paclitaxel-eluting balloons for femoropopliteal artery disease: evidence from a multicenter registry. *JACC Cardiovasc. Interv.* 6 282–289. 10.1016/j.jcin.2013.01.128 23517840

[B37] Mogosanu G. D. Grumezescu A. M. Chifiriuc M. C. (2014). Keratin-based biomaterials for biomedical applications. *Curr. Drug Targets* 15 518–530. 10.2174/1389450115666140307154143 24606008

[B38] Morice M. C. Serruys P. W. Sousa J. E. Fajadet J. Hayashi E. B. Perin M. (2002). A randomized comparison of a sirolimus-eluting stent with a standard stent for coronary revascularization. *N. Engl. J. Med.* 346 1773–1780. 10.1056/NEJMoa012843 12050336

[B39] Navarese E. P. Tandjung K. Claessen B. Andreotti F. Kowalewski M. Kandzari D. E. (2013). Safety and efficacy outcomes of first and second generation durable polymer drug eluting stents and biodegradable polymer biolimus eluting stents in clinical practice: comprehensive network meta-analysis. *BMJ* 347:f6530. 10.1136/bmj.f6530 24196498 PMC3819044

[B40] Nikanorov A. Smouse H. B. Osman K. Bialas M. Shrivastava S. Schwartz L. B. (2008). Fracture of self-expanding nitinol stents stressed in vitro under simulated intravascular conditions. *J. Vasc. Surg.* 48 435–440. 10.1016/j.jvs.2008.02.029 18486426

[B41] Nunez F. A. Callahan M. F. Trach S. Burnett L. R. Kislukhin V. Smith T. L. (2013). Hemodynamic recovery after hypovolemic shock with lactated Ringer’s and keratin resuscitation fluid (KRF), a novel colloid. *Artif. Cells Nanomed. Biotechnol.* 41 293–303. 10.3109/21691401.2012.747533 23305143

[B42] Oberhoff M. Herdeg C. Baumbach A. Karsch K. R. (2002). Stent-based antirestenotic coatings (sirolimus/paclitaxel). *Catheter. Cardiovasc. Interv.* 55 404–408. 10.1002/ccd.10034 11870952

[B43] Pace L. A. Plate J. F. Mannava S. Barnwell J. C. Koman L. A. Li Z. (2014). A human hair keratin hydrogel scaffold enhances median nerve regeneration in nonhuman primates: an electrophysiological and histological study. *Tissue Eng. Part A* 20 507–517. 10.1089/ten.TEA.2013.0084 24083825 PMC3926186

[B44] Park M. Shin H. K. Kim B. S. Kim M. J. Kim I. S. Park B. Y. (2015). Effect of discarded keratin-based biocomposite hydrogels on the wound healing process in vivo. *Mater. Sci. Eng. C* 55 88–94. 10.1016/j.msec.2015.03.033 26117742

[B45] Poranki D. Whitener W. Howse S. Mesen T. Howse E. Burnell J. (2014). Evaluation of skin regeneration after burns in vivo and rescue of cells after thermal stress in vitro following treatment with a keratin biomaterial. *J. Biomater. Appl.* 29 26–35. 10.1177/0885328213513310 24272161

[B46] Poranki D. R. Van Dyke M. E. (2014). The effect of gamma keratose on cell viability in vitro after thermal stress and the regulation of cell death pathway-specific gene expression. *Biomaterials* 35 4646–4655. 10.1016/j.biomaterials.2014.02.044 24631248

[B47] Rezazadeh M. Emami J. Mostafavi A. Rostami M. Hassanzadeh F. Sadeghi H. (2015). A rapid and sensitive HPLC method for quantitation of paclitaxel in biological samples using liquid-liquid extraction and UV detection: application to pharmacokinetics and tissues distribution study of paclitaxel loaded targeted polymeric micelles in tumor bearing mice. *J. Pharm. Pharm. Sci.* 18 647–660. 10.18433/J3RP6Z 26670364

[B48] Ricco J. B. Thanh Phong L. Schneider F. Illuminati G. Belmonte R. Valagier A. (2013). The diabetic foot: a review. *J. Cardiovasc. Surg.* 54 755–762. 24126512

[B49] Rits J. Van Herwaarden J. A. Jahrome A. K. Krievins D. Moll F. L. (2008). The incidence of arterial stent fractures with exclusion of coronary, aortic, and non-arterial settings. *Eur. J. Vasc. Endovasc. Surg.* 36 339–345. 10.1016/j.ejvs.2008.05.005 18602847

[B50] Rosenfield K. Jaff M. R. White C. J. Rocha-Singh K. Mena-Hurtado C. Metzger D. C. (2015). Trial of a paclitaxel-coated balloon for femoropopliteal artery disease. *N. Engl. J. Med.* 373 145–153. 10.1056/NEJMoa1406235 26106946

[B51] Rowinsky E. K. (1993). Clinical pharmacology of Taxol. *J. Natl. Cancer Inst. Monogr.* 15 25–37. 7912527

[B52] Saul J. M. Ellenburg M. D. De Guzman R. C. Van Dyke M. (2011). Keratin hydrogels support the sustained release of bioactive ciprofloxacin. *J. Biomed. Mater. Res. A* 98 544–553. 10.1002/jbm.a.33147 21681948

[B53] Scheinert D. Duda S. Zeller T. Krankenberg H. Ricke J. Bosiers M. (2014). The LEVANT I (Lutonix paclitaxel-coated balloon for the prevention of femoropopliteal restenosis) trial for femoropopliteal revascularization: first-in-human randomized trial of low-dose drug-coated balloon versus uncoated balloon angioplasty. *JACC Cardiovasc. Interv.* 7 10–19. 10.1016/j.jcin.2013.05.022 24456716

[B54] Scheinert D. Scheinert S. Sax J. Piorkowski C. Braunlich S. Ulrich M. (2005). Prevalence and clinical impact of stent fractures after femoropopliteal stenting. *J. Am. Coll. Cardiol.* 45 312–315. 10.1016/j.jacc.2004.11.026 15653033

[B55] Scheller B. Speck U. Abramjuk C. Bernhardt U. Bohm M. Nickenig G. (2004). Paclitaxel balloon coating, a novel method for prevention and therapy of restenosis. *Circulation* 110 810–814. 10.1161/01.CIR.0000138929.71660.E0 15302790

[B56] Scheller B. Speck U. Romeike B. Schmitt A. Sovak M. Böhm M. (2003a). Contrast media as carriers for local drug delivery: successful inhibition of neointimal proliferation in the porcine coronary stent model. *Eur. Heart J.* 24 1462–1467. 12909076 10.1016/s0195-668x(03)00317-8

[B57] Scheller B. Speck U. Schmitt A. Böhm M. Nickenig G. (2003b). Addition of paclitaxel to contrast media prevents restenosis after coronary stent implantation. *J. Am. Coll. Cardiol.* 42 1415–1420. 14563585 10.1016/s0735-1097(03)01056-8

[B58] Schmidt A. Piorkowski M. Gorner H. Steiner S. Bausback Y. Scheinert S. (2016). Drug-coated balloons for complex femoropopliteal lesions: 2-year results of a real-world registry. *JACC Cardiovasc. Interv.* 9 715–724. 10.1016/j.jcin.2015.12.267 27056311

[B59] Schroeder H. Meyer D. R. Lux B. Ruecker F. Martorana M. Duda S. (2015). Two-year results of a low-dose drug-coated balloon for revascularization of the femoropopliteal artery: outcomes from the ILLUMENATE first-in-human study. *Catheter. Cardiovasc. Interv.* 86 278–286. 10.1002/ccd.25900 25708850 PMC6585947

[B60] Seidlitz A. Kotzan N. Nagel S. Reske T. Grabow N. Harder C. (2013). In vitro determination of drug transfer from drug-coated balloons. *PLoS One* 8:e83992. 10.1371/journal.pone.0083992 24391863 PMC3877149

[B61] Shen D. Wang X. Zhang L. Zhao X. Li J. Cheng K. (2011). The amelioration of cardiac dysfunction after myocardial infarction by the injection of keratin biomaterials derived from human hair. *Biomaterials* 32 9290–9299. 10.1016/j.biomaterials.2011.08.057 21885119

[B62] Sierpinski P. Garrett J. Ma J. Apel P. Klorig D. Smith T. (2008). The use of keratin biomaterials derived from human hair for the promotion of rapid regeneration of peripheral nerves. *Biomaterials* 29 118–128. 10.1016/j.biomaterials.2007.08.023 17919720

[B63] Singh G. D. Armstrong E. J. Laird J. R. (2014). Femoropopliteal in-stent restenosis: current treatment strategies. *J. Cardiovasc. Surg.* 55 325–333. 24755700

[B64] Speck U. Cremers B. Kelsch B. Biedermann M. Clever Y. P. Schaffner S. (2012). Do pharmacokinetics explain persistent restenosis inhibition by a single dose of paclitaxel? *Circ. Cardiovasc. Interv.* 5 392–400. 10.1161/CIRCINTERVENTIONS.111.967794 22619258

[B65] Spectranetics (2017). *Stellarex DCB with EnduraCoat Technology.* Colorado Springs, CO: Spectranetics.

[B66] Stone G. W. Ellis S. G. Cox D. A. Hermiller J. O’shaughnessy C. Mann J. T. (2004). A polymer-based, paclitaxel-eluting stent in patients with coronary artery disease. *N. Engl. J. Med.* 350 221–231. 10.1056/NEJMoa032441 14724301

[B67] Stone G. W. Midei M. Newman W. Sanz M. Hermiller J. B. Williams J. (2009). Randomized comparison of everolimus-eluting and paclitaxel-eluting stents: two-year clinical follow-up from the Clinical Evaluation of the Xience V Everolimus Eluting Coronary Stent System in the Treatment of Patients with de novo Native Coronary Artery Lesions (SPIRIT) III trial. *Circulation* 119 680–686. 10.1161/CIRCULATIONAHA.108.803528 19171853

[B68] Suffness M. (1995). *TAXOL Science and Applications.* Boca Raton, FL: CRC Press.

[B69] Sumpio B. E. Lee T. Blume P. A. (2003). Vascular evaluation and arterial reconstruction of the diabetic foot. *Clin. Podiatr. Med. Surg.* 20 689–708. 10.1016/S0891-8422(03)00088-0 14636033

[B70] Tarr B. D. Yalkowsky S. H. (1987). A new parenteral vehicle for the administration of some poorly water soluble anti-cancer drugs. *J. Parenteral. Sci. Technol.* 41 31–33. 3559832

[B71] Tekade R. K. D’emanuele A. Elhissi A. Agrawal A. Jain A. Arafat B. T. (2013). Extraction and RP-HPLC determination of Taxol in rat plasma, cell culture and quality control samples. *J. Biomed. Res.* 27 394–405. 10.7555/JBR.27.20120123 24086173 PMC3783825

[B72] Tepe G. Laird J. Schneider P. Brodmann M. Krishnan P. Micari A. (2015a). Drug-coated balloon versus standard percutaneous transluminal angioplasty for the treatment of superficial femoral and popliteal peripheral artery disease: 12-month results from the IN/PACT SFA randomized trial. *Circulation* 131 495–502. 10.1161/CIRCULATIONAHA.114.011004 25472980 PMC4323569

[B73] Tepe G. Schnorr B. Albrecht T. Brechtel K. Claussen C. D. Scheller B. (2015b). Angioplasty of femoral-popliteal arteries with drug-coated balloons: 5-year follow-up of the THUNDER trial. *JACC Cardiovasc. Interv.* 8 102–108. 10.1016/j.jcin.2014.07.023 25616822

[B74] Tepe G. Zeller T. Albrecht T. Heller S. Schwarzwälder U. Beregi J. (2008). Local delivery of paclitaxel to inhibit restenosis during angioplasty of the leg. *N. Engl. J. Med.* 358 689–699. 10.1056/NEJMoa0706356 18272892

[B75] Turner E. A. Stenson A. C. Yazdani S. K. (2017). HPLC-MS/MS method for quantification of paclitaxel from keratin containing samples. *J. Pharm. Biomed. Anal.* 139 247–251. 10.1016/j.jpba.2017.03.011 28324728 PMC5410662

[B76] Venkatasubbu G. D. Ramasamy S. Avadhani G. S. Ramakrishnan V. Kumar J. (2012). Surface modification and paclitaxel drug delivery of folic acid modified polyethylene glycol functionalized hydroxyapatite nanoparticles. *Powder Technol.* 235 437–442. 10.1016/j.powtec.2012.11.003

[B77] Werk M. Albrecht T. Meyer D. R. Ahmed M. N. Behne A. Dietz U. (2012). Paclitaxel-coated balloons reduce restenosis after femoro-popliteal angioplasty: evidence from the randomized PACIFIER trial. *Circ. Cardiovasc. Interv.* 5 831–840. 10.1161/CIRCINTERVENTIONS.112.971630 23192918

[B78] Werk M. Langner S. Reinkensmeier B. Boettcher H. F. Tepe G. Dietz U. (2008). Inhibition of restenosis in femoropopliteal arteries: paclitaxel-coated versus uncoated balloon: femoral paclitaxel randomized pilot trial. *Circulation* 118 1358–1365. 10.1161/CIRCULATIONAHA.107.735985 18779447

[B79] Yazdani S. K. Berry J. L. (2009). Development of an in vitro system to assess stent-induced smooth muscle cell proliferation: a feasibility study. *J. Vasc. Interv. Radiol.* 20 101–106. 10.1016/j.jvir.2008.09.025 19028120

[B80] Yazdani S. K. Pacheco E. Nakano M. Otsuka F. Naisbitt S. Kolodgie F. D. (2014). Vascular, downstream, and pharmacokinetic responses to treatment with a low dose drug-coated balloon in a swine femoral artery model. *Catheter. Cardiovasc. Interv.* 83 132–140. 10.1002/ccd.24995 23703778

[B81] Zeller T. Baumgartner I. Scheinert D. Brodmann M. Bosiers M. Micari A. (2014). Drug-eluting balloon versus standard balloon angioplasty for infrapopliteal arterial revascularization in critical limb ischemia: 12-month results from the IN/PACT DEEP randomized trial. *J. Am. Coll. Cardiol.* 64 1568–1576. 10.1016/j.jacc.2014.06.1198 25301459

[B82] Zhang J. Xu X. Kong J. Xu R. Fan X. Chen J. (2017). Systematic review and meta-analysis of drug-eluting balloon and stent for infrapopliteal artery revascularization. *Vasc. Endovascular. Surg.* 51 72–83. 10.1177/1538574416689426 28103754

[B83] Zhang Y. L. Bendrick-Peart J. Strom T. Haschke M. Christians U. (2005). Development and validation of a high-throughput assay for quantification of the proliferation inhibitor ABT-578 using LC/LC-MS/MS in blood and tissue samples. *Ther. Drug Monit.* 27 770–778. 10.1097/01.ftd.0000185766.52126.bd 16306853

